# MEMS reservoir computing system with stiffness modulation for multi-scene data processing at the edge

**DOI:** 10.1038/s41378-024-00701-9

**Published:** 2024-06-24

**Authors:** Xiaowei Guo, Wuhao Yang, Xingyin Xiong, Zheng Wang, Xudong Zou

**Affiliations:** 1 The State Key Laboratory of Transducer Technology, Aerospace Information Research Institute, Chinese Academy of Sciences, Beijing, China; 2 School of Electronic, Electrical and Communication Engineering, University of Chinese Academy of Sciences, Beijing, China; 3 QILU Aerospace Information Research Institute, Jinan, China

**Keywords:** Engineering, Electrical and electronic engineering

## Abstract

Reservoir computing (RC) is a bio-inspired neural network structure which can be implemented in hardware with ease. It has been applied across various fields such as memristors, and electrochemical reactions, among which the micro-electro-mechanical systems (MEMS) is supposed to be the closest to sensing and computing integration. While previous MEMS RCs have demonstrated their potential as reservoirs, the amplitude modulation mode was found to be inadequate for computing directly upon sensing. To achieve this objective, this paper introduces a novel MEMS reservoir computing system based on stiffness modulation, where natural signals directly influence the system stiffness as input. Under this innovative concept, information can be processed locally without the need for advanced data collection and pre-processing. We present an integrated RC system characterized by small volume and low power consumption, eliminating complicated setups in traditional MEMS RC for data discretization and transduction. Both simulation and experiment were conducted on our accelerometer. We performed nonlinearity tuning for the resonator and optimized the post-processing algorithm by introducing a digital mask operator. Consequently, our MEMS RC is capable of both classification and forecasting, surpassing the capabilities of our previous non-delay-based architecture. Our method successfully processed word classification, with a 99.8% accuracy, and chaos forecasting, with a 0.0305 normalized mean square error (NMSE), demonstrating its adaptability for multi-scene data processing. This work is essential as it presents a novel MEMS RC with stiffness modulation, offering a simplified, efficient approach to integrate sensing and computing. Our approach has initiated edge computing, enabling emergent applications in MEMS for local computations.

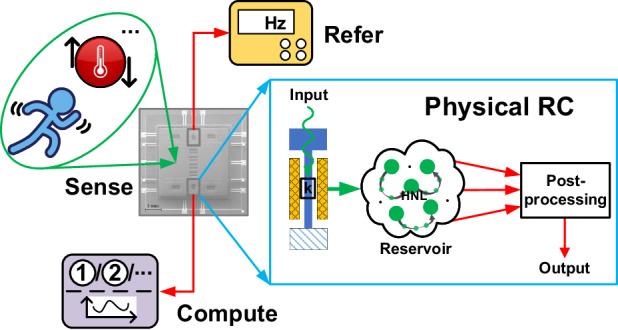

## Introduction

With the booming growth of the Internet of Things (IoT) in our information-driven society, massive raw data generated by thousands of sensor nodes is increasingly consuming transmission capacity. This necessitates the ability of systems to process information efficiently with low power consumption. Therefore, the localization of signal processing is highly desired, where sensing and intelligence need to be integrated, achieving edge computing. Reservoir computing (RC), inspired by recurrent neural networks (RNN), stands out as the best-in-class solution for dealing with time-series data and is well-suited for physical implementation^1^. It has a simple algorithmic structure, and the reservoir can be realized by nonlinear devices. Consequently, researchers have dedicated themselves to physical RC in recent years^2–5^. However, most of them remained in the verification stage, proving the feasibility of devices acting as a reservoir for computing but losing sight of sensing. Additionally, some RC systems often have a large volume and complicated setup, such as optical RC^6^. These studies primarily explore the potential of devices to function as a reservoir, leveraging their inherent nonlinearity and fading memory crucial for RC. However, they often overlook the direct processing of sensor data. This aspect, vital for real-time data interpretation, is not adequately addressed in their methodologies.

Micro-electro-mechanical systems (MEMS) take advantages of small volume, low power consumption^7–9^. More importantly, they are primarily designed for sensors, bringing MEMS RC closer to the original intention of edge computing. Previous researches relating to MEMS RC also only verified its feasibility^10–12^, but failed to provide an integrated sensor system combining sensing and computing. Information was injected as a dataset and then modulated to the drive voltage by amplitude modulation. The sensing characteristic of MEMS was neglected, focusing only on the computing characteristic. Moreover, the three layers (input layer, reservoir layer, and output layer) were always set up separately at the hardware level, resulting in a discrete system with a large volume. Some improvements have been proposed, such as using bias time multiplexing to divide input and mask^13,14^, using hybrid nonlinearity (HNL) to enhance RC ability for classification tasks^15,16^, and using structural design to obtain MEMS neurons^17,18^. However, drawbacks need to be considered, such as feedback still existing, resulting in a separate system, poor long-term memory capacity (MC), and pending tasks have to be designed (basically simple classification tasks), especially for the using device, respectively. Most works did not obtain a system fully suitable for edge computing dealing with various scenes.

**Fig. 1:**
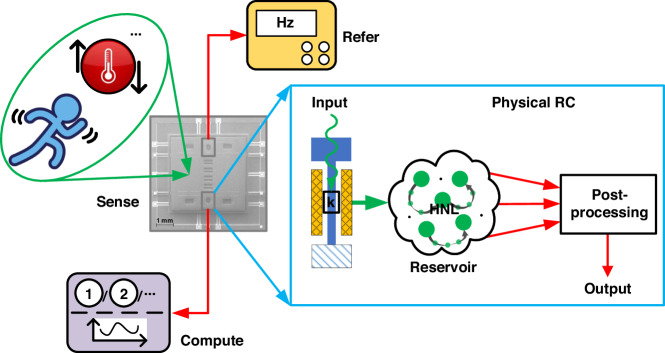
Concept schematic of stiffness-modulated MEMS RC. Natural signals directly affect the stiffness k of the MEMS resonant accelerometer, and then the response is collected and sent to the algorithm, which is often a simple linear regression. It makes use of the resonator HNL to obtain a rich reservoir. This architecture can preliminarily integrate sensing and computing in a single device

This paper provides an integrated MEMS RC based on stiffness modulation, as shown in Fig. 1. The novel architecture can be applied to a differential MEMS resonant accelerometer (also other kind of resonant sensors). It utilizes stiffness modulation, where the input is sensed and injected as a natural signal to disturb the stiffness k of the resonator. The rich reservoir states generated by HNL are then collected by integrated circuits (IC) and field programmable gate array (FPGA) for computing. The objective of this study is to address the previously overlooked aspect of direct sensor data processing, while preserving the advanced computational capabilities of MEMS RC. We intended to achieve a fusion of sensing and intelligence across multiple scenarios. Our method introduces a new sensing paradigm with direct and local data processing, eliminating the need for conventional frequency monitoring or references. We cleared away data discretization between the first two layers, reducing system complexity. This way, information from natural signals (e.g., acceleration or temperature) can be directly processed by the RC system at the edge. We also optimized the algorithm in the third layer to address shortcomings in the forecasting task within our original architecture^15^, while maintaining good performance in classification tasks, presenting a MEMS RC effective in multi-scene applications. This research aims to contribute to the development of a new generation of intelligent sensors and sensor systems. The method not only addresses the gap in integrating sensing with computing for edge applications, but also presents a compact, energy-efficient solution for data processing in various scenarios, showcasing its relevance and novelty in intelligent MEMS sensor technology.

## Results

### MEMS RC with stiffness modulation

In our stiffness-modulated MEMS RC, the reservoir states are obtained by HNL of the nonlinear resonator which acts as the reservoir layer, where a comprehensive Duffing function is given by:

1 $${m}_{{{\mathrm{eff}}}}\ddot{x}+\frac{{m}_{{{\mathrm{eff}}}}{\omega }_{0}}{Q}\dot{x}+{k}_{1}x+{k}_{3}{x}^{3}=\frac{{C}_{0}{d}_{0}}{2{\left({d}_{0}-x\right)}^{2}}{\left({V}_{{{\mathrm{dc}}}}-{V}_{{{\mathrm{ac}}}}\cos \left(\varOmega t\right)\right)}^{2}-\frac{{C}_{0}{d}_{0}}{2{\left({d}_{0}+x\right)}^{2}}{{V}_{b}}^{2}$$

where m_eff_, x, Q, ω_0_, k_1_, and k_3_ are the effective lumped mass, displacement of the silicon beam, quality factor, natural resonant frequency, linear mechanical stiffness, and nonlinear mechanical stiffness, respectively, and C_0_, d_0_, V_dc_, V_ac_, and Ω denote the initial capacitance, initial gap of the parallel plate electrode, bias voltage, drive voltage, and drive frequency, respectively. Previous MEMC RCs utilized amplitude modulation, modulating the input signal to V_ac_ electrically with x as the electrically measured output response^10,12^. By ADC and DAC, the response is added to the input through a delay-loop with a certain time interval determined by the mask operation. This intricate process, coupled with the transduction between electricity and force, escalates the system’s complexity and error rate. To address these issues, we have introduced a stiffness modulation method, injecting the input into the k_1_ term via natural signals. This approach allows the stiffness disturbance to convey information, directly impacting the system response x. In this way, the drive force is fixed and no transduction is needed, and thus streamlining the RC system. This not only simplifies the system but also enables it to concurrently sense mechanical signals and perform related computational tasks. Our novel modulation technique circumvents the need for energy conversion from electricity to force—a frequent requirement in amplitude-modulated RC systems where a dataset must first be collected and then converted into electrostatic force.

**Fig. 2:**
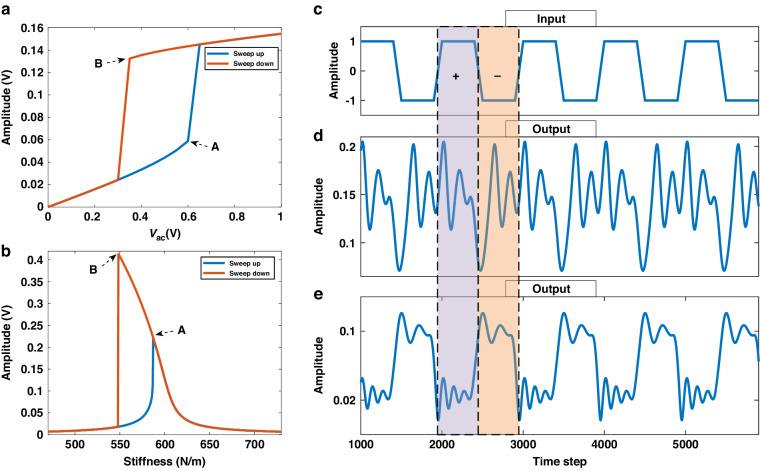
Different nonlinear responses of MEMS resonator. **a** Amplitude-amplitude nonlinear response. **b** Stiffness-amplitude nonlinear response. Note that we usually choose point A as the operation point. **c** Input data series. **d** Output of amplitude-amplitude nonlinearity. **e** Output of stiffness-amplitude nonlinearity. It shows that the response of out stiffness modulation mode can distinguish bidirectional data better

Considering the pivotal roles of nonlinearity and MC in RC, it is imperative to precisely define these two characteristics within our architecture. In the operation of a nonlinear resonator, HNL occurs, which contains two kinds of nonlinearities: Duffing nonlinearity (DNL) and transient nonlinearity (TNL). DNL is instrumental in creating a robust nonlinear mapping, and TNL is essential for imparting a fading characteristic. Amplitude-modulated RC capitalizes on the amplitude-amplitude nonlinear response, as shown in Fig. 2a, while stiffness-modulated RC leverages the stiffness-amplitude nonlinear response, illustrated in Fig. 2b, both emanating from the HNL. In comparison, the stiffness modulation mode exhibits a more distinctive nonlinearity shape, incorporating inflections that facilitate more effective mapping of data into high-dimensional space. Point A and point B are bifurcation points where the dynamics is more abundant, from which we often choose as operation points. In this study, we controlled the input acceleration signal (bidirectional) to maintain the stiffness within a certain range around point A for better performance, and optimized with the RC algorithm through nonlinearity tuning^16^. Another merit of the stiffness modulation mode is that it can process bidirectional data. As Fig. 2c demonstrates, positive and negative data can be distinctly identified since stiffness either increases or decreases, in contrast to the amplitude modulation mode, where data is subjected to absolute value transformation. Figure 2d, e showcases the output of the resonator under both modes, clearly indicating that bidirectional input is more effectively segregated in the stiffness modulation mode. As TNL can usually suffice for MC, particularly for short-term applications, classification tasks demand robust nearby coupling, thus rendering short-term MC adequate for high performance. However, forecasting tasks typically require long-term time dependencies. To facilitate this, we retained the delay-loop for long-term MC, but implemented it digitally in the output layer, resulting in improved performance due to reduced noise. Further optimization of the algorithm is elaborated in the subsequent section.

### Optimization for forecasting task

**Fig. 3:**
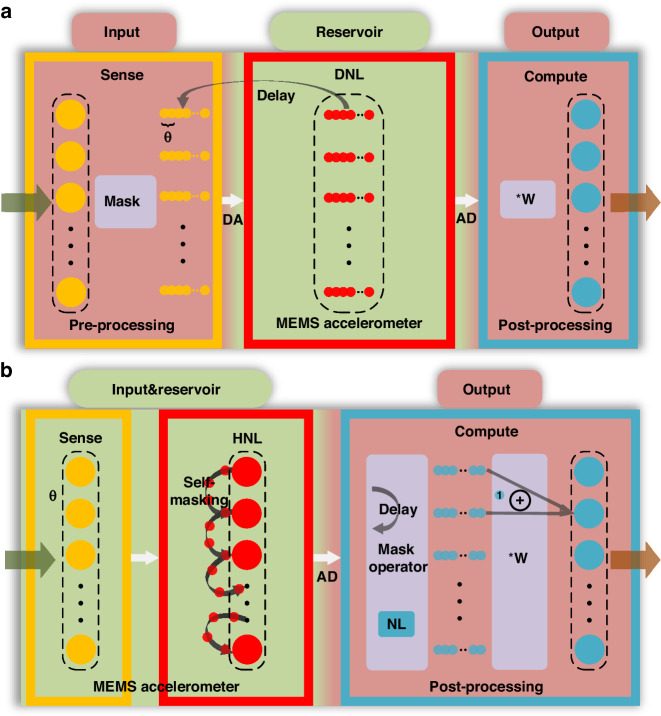
Comparison of MEMS RC architectures. **a** Traditional amplitude-modulated MEMS RC. The three layers are separated and mainly DNL is used. **b** Optimized stiffness-modulated MEMS RC. Self-masking effect derived from HNL is used. Analog information is seamlessly sensed and directly injected into RC. The digital mask operator contains a mask, delay, and nonlinear nodes

We first compare the traditional amplitude-modulated MEMS RC architecture as displayed in Fig. 3a. The input layer and output layer both reside in the digital domain (red part), while the reservoir layer exists in the analog domain (green part). This segmented architecture necessitates a DAC between the input data and the physical reservoir due to the delay between the analog and digital domains. Fundamentally, this approach utilizes the DNL of the resonator and primarily derives MC from the delay mechanism. Additionally, an ADC is essential for sampling the output response and channeling it into the regression process. Further elaboration on important parameters is provided in the subsequent structure description. As we can see, the traditional RC is unable to directly process natural signals without first collecting and converting them into digital form. This requirement increases system complexity and power consumption and, furthermore, fails to achieve an integration of sensing and intelligence.

Our stiffness-modulated MEMS RC architecture is shown in Fig. 3b. The input and reservoir layers are combined in the analog domain. Here, data is directly injected into the MEMS accelerometer as a natural signal, consequently influencing the stiffness. Our reservoir states capitalize on HNL, which introduces a self-masking effect. By nonlinearity tuning with the driving voltage and the time interval θ, a dynamic coupling is established between adjacent data points, yielding a rich response. This nonlinear transformation with only HNL is suitable for classification tasks, but falls short in handling complex forecasting tasks. Therefore, we proposed a digital mask operator in the digital domain which colligates mask, delay, and nonlinear nodes (the NL block). This is tantamount to the analogous component in conventional delay-based RC algorithms but is implemented digitally following the resonator’s response sampling by ADC. We note that θ in our architecture represents the sampling time rather than the input time interval, as the input is analog, with each data point in the analog domain being a presumed sampled point. To navigate the relationship between θ and RC properties, it’s crucial to optimize both the total duration of data and the number of expected output points. In the mask operator, the mask is a vector of length N, randomly set within a range of −1 to 1. The delay, with a length of τ, determines the temporal relevance of past data to the current data, thereby enhancing the system MC. We incorporated three nonlinear nodes to enrich the feature: a quadratic node (self-multiplied), a recurrent node (multiplied by previous data), and a Sigmoid node (processed by a Sigmoid function)^19^. Specifically, the quadratic node introduces self-nonlinearity, the recurrent node provides local feedback, and the Sigmoid node, positioned before the digital delay-loop, primarily serves as an active function for rescaling. The sampled response first passes through the first two nodes in parallel, and then the resulting three data streams are multiplied by the mask, producing a 3N vector, which subsequently passes through the Sigmoid node. For long-term MC acquisition, we concatenated the current vector with several preceding vectors^20^. A selection of elements is maintained to strike a balance between speed and precision, also mitigating the risk of overfitting. During training, a bias term is added to bolster performance. The training method employed is ridge regression, that only a weight vector **w** is needed to be trained. Ridge regression addresses multicollinearity in linear regression by adding an L2 penalty term, which shrinks coefficients to stabilize the model and prevent overfitting. Further details on the RC algorithm equations and signal flows pertinent to different tasks are available in the “Materials and Methods” section. This architecture has adjustable parameters that both forecasting tasks and classification tasks can be handled.

### Physical RC implementation

**Fig. 4:**
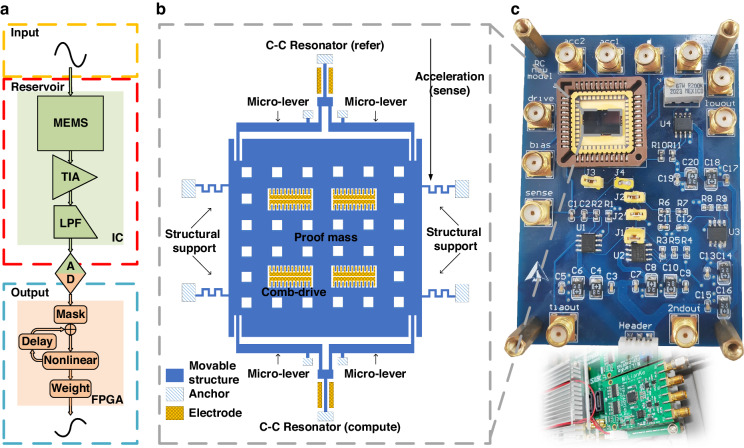
Hardware architecture of MEMS RC system. **a** Schematic of our hardware system with integration of MEMS, IC, and FPGA. The first two layers are combined, while the third layer is connected by an ADC. **b** Schematic of the used differential MEMS resonant accelerometer. Two resonators are connected by a proof mass on which several comb-drives act as the tuning structure. **c** Actual images of IC and FPGA

We applied the new architecture to a differential MEMS resonant accelerometer^8^, and provided an RC system that integrates MEMS, IC, and FPGA, thereby realizing a physical RC. The schematic of our hardware system is depicted in Fig. 4a. The analog signal is directly fed into the MEMS without pre-processing, and its response is captured by the interface circuit. The resonator displacement is detected as a current by capacitive detection, subsequently amplified to a voltage by a transimpedance amplifier (TIA). It is further amplified by a secondary amplifier for gain control. A Lowpass filter (LPF) demodulates high-frequency signals to extract the information of interest, which is then sampled by an ADC. Additionally, there is a module on the IC, operating in parallel with the MEMS accelerometer, to minimize feed-through. The algorithmic processing of the output is conducted within interaction codes. The design of the accelerometer is illustrated in Fig. 4b. It features two double-ended tuning fork resonators, each connected at one end to a proof mass through a pair of micro-levers. Upon sensing acceleration, the top resonator functions as the reference module, while the bottom resonator serves as the computing module. The dimensions of the computing resonator are 400 μm in length, 6 μm in width, and 50 μm in thickness, with its natural resonant frequency simulated to be around 180.15 kHz. There are also several comb-drives in the center for tuning purposes. Details on device fabrication can be found in “Materials and methods”. Figure 4c showcases actual images of the used IC and FPGA. The MEMS was inserted on IC, and only a portion of the FPGA was employed, encompassing the ADC and several computational modules.

**Fig. 5:**
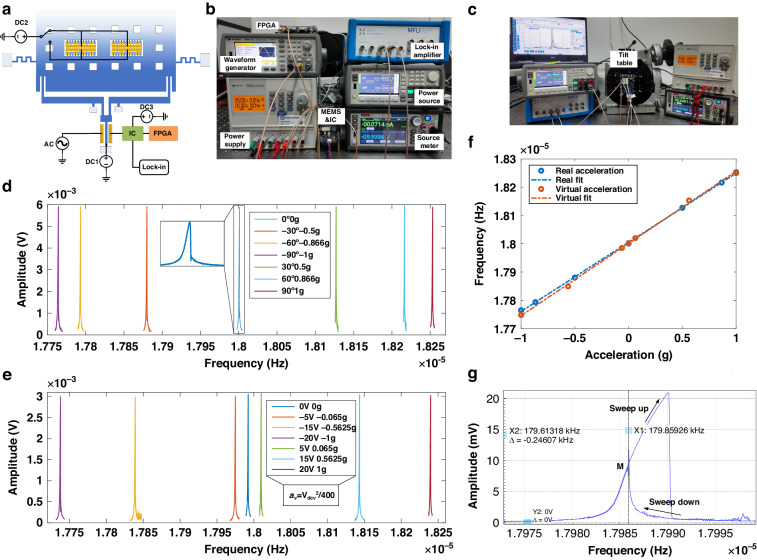
Experiment setups and device characteristics. **a** Schematic of the test circuit. **b** Image of experiment setups for task processing. **c** Image of experiment setups for device calibration. **d** Frequency responses varying with real accelerations. **e** Frequency responses varying with virtual accelerations. **f** Calibration results with a linear fit. The scale factors are 2447 Hz/g and 2558 Hz/g, respectively. **g** Nonlinear frequency response

The schematic of the test circuit is shown in Fig. 5a. We pay attention to the computing resonator, which is driven by a bias V_dc_, and a drive V_ac_ with a frequency f_d_. The reference resonator can operate within our close-loop circuit^21^. Figure 5b, c display images of our experiment setups dedicated to task processing and device calibration, respectively. The IC was powered by a power supply (KEYSIGHT U8032A), on which the MEMS accelerometer was connected to a source meter (KEITHLEY 2450) for V_dc_, a waveform generator (KEYSIGHT 33600 A) for V_ac_, and a lock-in amplifier (MFLI 500 kHz/5 MHz) for preliminary characterization. Given that many datasets in the RC field are associated with biological collection or chaotic systems, there is no natural signal which can fully represents these data. So, to utilize stiffness modulation, an electrostatic force varying with input data was applied to the accelerometer to simulate a varying acceleration series. For this purpose, we connected the comb-drives to a power source (KEYSIGHT B2962A), providing a voltage V_dcv_ and thus generating virtual acceleration, as shown in Fig. 5b. This voltage was programmed for various tasks, and the relationship between V_dcv_ and virtual acceleration a_v_ is given by:

2 $${a}_{v}={k}_{v}{{V}_{{dcv}}}^{2}$$

where k_v_ is the conversion coefficient^22^. In this manner, the accelerometer receives the predetermined acceleration signals via stiffness disturbance, enabling the operation of our stiffness-modulated MEMS RC. Finally, the output response was collected by the FPGA. Post-processing operations were conducted in tandem with the feeding of information into the system. In order to calibrate our accelerometer, we also used a tilt table to provide real acceleration, as demonstrated in Fig. 5c, and compared the two input methods. Other setups were basically the same.

We now present the characteristics of our MEMS accelerometer. We both set V_dc_ = 10 V, V_ac_ = 3 mV for the two methods, and the sets of frequency responses are displayed in Fig. 5d for the real acceleration method, and Fig. 5e for the virtual acceleration method. The resonance is about 180.02 kHz at zero-bias, accompanied by a slight nonlinear curve shape. Through calibration, we determined k_v_ = 0.0025 g/V^2^, and around 20 V corresponding to 1 g. Figure 5f illustrates the frequency-acceleration characteristic of both methods, with scale factors of 2447 Hz/g for the real one, and 2558 Hz/g for the virtual one, both fairly linear. This minor difference was taken into account while programming the input data for the power source, that during implementation, a conversion coefficient of 2447/2558 is applied to ensure that the frequency change corresponding to a “virtual 1 g” aligns with that of a “real 1 g”. Finally, we set V_dc_ = 20 V, V_ac_ = 10 mV to induce greater nonlinearity and conducted a bidirectional frequency sweep, as shown in Fig. 5g. We got two bifurcation points as expected, with the smaller one, point M, located around 179.86 kHz, near which we selected the operation point. For principles of operation point tuning, refer to the method section.

### Word classification

**Fig. 6:**
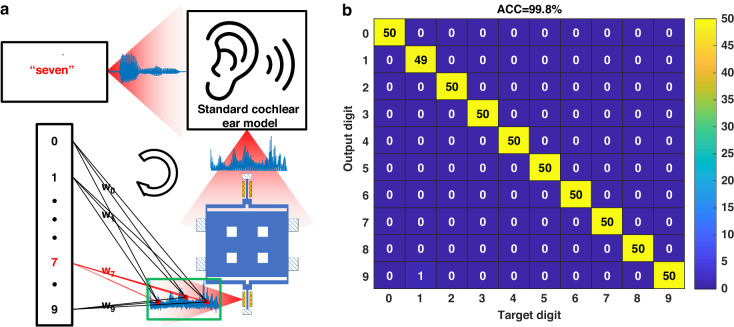
TI-46 result. **a** Schematic diagram of the MEMS RC processing system of the TI-46 task. The voice signals go through a standard cochlear ear model and are mapped by the reservoir. The winner-takes-all approach decides the final output digit. **b** Confusion matrix for classifying the ten digits. The best accuracy was 99.8% with tenfold cross-validation

We first evaluated our novel concept using a speech word classification task, specifically the TI-46 dataset^23^. The dataset includes ten spoken digits (0–9), each pronounced ten times by five different female speakers. To enhance generalization, we employed a tenfold cross-validation method, given the limited number of samples, utilizing 450 words for training and 50 words for testing, repeated ten times. Each word is sampled at 12.5 kHz with variable time length. As shown in Fig. 6a, we pre-processed the input by using the standard cochlear ear model^24^, a prevalent technique for extracting acoustic features. The sample rate of the ADC after the first two layers was set to 1.25 MHz, in accordance with the Nyquist Sampling Theorem, and the required data points were down-sampled at a rate of 1/θ. The mask operator was executed in FPGA (green box) for post-processing, while maintaining the physical RC as a non-delay structure. In the hardware setup, we set V_dc_ = 10 V, V_ac_ = 1 V, f_d_ = 180.32 kHz for an optimum operation point as discussed in Fig. 2b, and time-dependent parameters θ = 0.2 T_d_ = 0.049 ms to strengthen data coupling for classification tasks. T_d_ = 2Q/ω_0_ is the decay time of the resonator, where ω_0_ is its natural frequency. We trained ten classifiers **w**_**0**_ ~ **w**_**9**_ for the different digits, with a target value of 1 when the input word matched the sought digit, and 0 otherwise. A winner-takes-all strategy was applied to determine the predicted digit, that we took the largest output value. Further details can be found in the Methods section for an in-depth explanation. Figure 6b shows the result as a confusion matrix. Our system achieved a 99.8% accuracy, surpassing the 95.7% accuracy of electronic systems^25^, 99.2% of memristor RC^26^, 99.6% of optoelectronic RC^3^, and matching the performance of our previous work utilizing a disjointed system^11^.

### Chaos forecasting

**Fig. 7:**
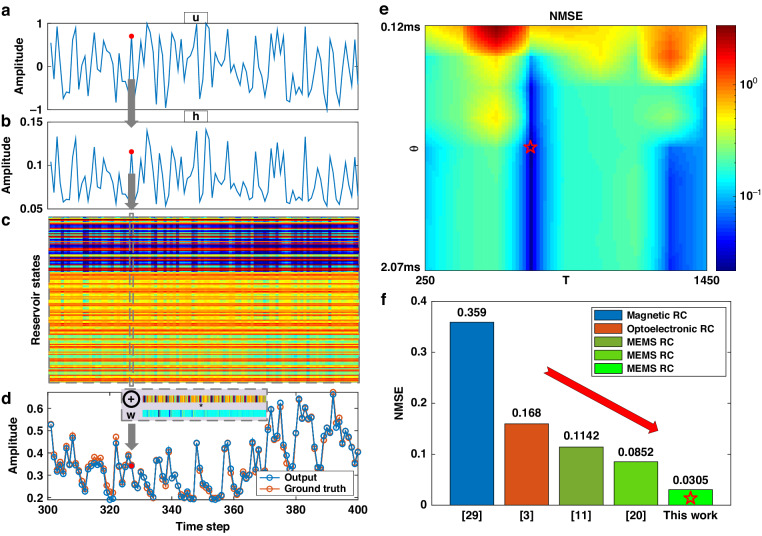
NARMA-10 result. **a** Normalized input data. **b** Data after mapped by the resonator. **c** Reservoir states after processed by the mask operator. Each column represents one time point. **d** Output after concatenation and weight comparing with the ground truth. These points exhibit a high degree of overlap, indicating a minimal error between the predicted and true values. **e** NMSE varying with θ and τ. **f** NMSE comparing with other works

To validate our method for multi-scene as well as our optimization, we tested the well-known NARMA-10 forecasting task in the RC community^27^. Chaos forecasting is often regarded as the hardest of the hard for machine learning, where most networks require a large number of meta-parameters^28^. The normalized original data (taking 100 points as an example) are shown in Fig. 7a. We set V_dc_ = 20 V for a bigger nonlinearity, and V_ac_ = 1 V, f_d_ = 179.86 kHz for a proper reservoir. Figure 7b shows the mapping data demodulated from the resonator response. After the mask operator, we obtained a reservoir states matrix, as shown in Fig. 7c. Each column represents the current states. It was then concatenated with previous columns and multiplied by the weight. The prediction output is compared with ground truth in Fig. 7d. It is worth mentioning that for forecasting tasks, a θ value several times greater than T_d_ is needed to avoid strong adjacent data coupling, yet a long coupling is required by the delay τ. We swept θ and τ as shown in Fig. 7e, achieving the lowest normalized mean square error (NMSE) of only 0.0305 when θ = 4.5 T_d_ = 1.1 ms and τ = 14N = 700. This indicates that the resonator response remains largely unchanged as θ exceeds several multiples of T_d_, resulting in comparable accuracy, but lacking sufficient long-term MC. Hence, under these conditions, the digital delay predominates, yielding optimal performance when feedback connects the fifth data point ahead to the current point. In Fig. 7f, two works of other types of physical RC^3,29^, and two of our previous works on amplitude-modulated MEMS RC^11,20^, are compared, underscoring the superiority of our novel architecture. The improved efficiency in our system is attributed to MEMS’s heightened sensitivity to mechanical over electrical signals, coupled with our enhancements to the output layer that boost the system’s long-term memory capacity, crucial for forecasting tasks. Taken together, these results suggest that our system delivers exceptional performance across various tasks, with a simpler structure than traditional RC.

## Discussion

This research proposes a novel MEMS reservoir computing system that co-localizes sensing and intelligence based on stiffness modulation. Natural signals, containing interested data, can be directly processed upon being sensed by MEMS accelerometer, so that data discretization and feedback from analog reservoir to digital input can be eliminated. The system has simple setup and small power consumption which integrates MEMS, IC, and FPGA. Leveraging nonlinearity tuning and algorithm optimization, it successfully processed classification task, as well as forecasting task which is thought hard for original non-delay RC architecture. In this research, the accuracy of TI-46 task is 99.8% and the NMSE of NARMA-10 task is 0.0305, both demonstrating superiority over other state-of-the-art works, compared to previous works, such as 99.6% in TI-46^3^, and 0.1142 in NARMA-10^11^. This work enhances the theoretical understanding of a novel modulation approach in MEMS Reservoir Computing, delving into the nonlinear dynamics and operational mechanics. It also contributes significantly to the advancement of the RC structure through algorithmic improvements, offering a deeper insight into the interplay between physical mechanisms and computational efficiency in MEMS technology.

Two possible explanations account for the enhanced performance. Firstly, stiffness modulation efficiently captures and broadcasts natural signals, especially inertial signals like acceleration, outperforming amplitude modulation. The resonator’s stiffness is directly influenced by inertia force without other transductions, obtaining abundant reservoir states. Amplitude modulation is limited to base band and capacitor driving, so data transformed from electrical signals may not be entirely “pure”. In other words, MEMS RC is more sensitive to mechanical signals than electrical signals at the hardware level. Secondly, optimization of the post-processing algorithm, including the addition of a mask operator with delay feedback and the concatenation of feature vectors, enhances long-term memory capacity (MC). Implemented in the digital domain, these processes are considered noiseless, thereby increasing accuracy. This contributes more to the RC algorithm at the software level.

The significance of this research lies in its groundbreaking concept of integrating sensing and computing within a MEMS RC, enhancing the efficiency and functionality of multi-scene IoT devices. Although the proposed MEMS RC has not yet been tested in a real-world acceleration or temperature application scenario due to non-wearable conditions, the virtual acceleration experiment demonstrates equivalent system capabilities. Moreover, our MEMS RC has been validated in two scenarios in our previous work: acceleration recognition of IMU motions^15,30^, and temperature compensation for MEMS resonators^20^. Our work advances the development of sensing-computing integration in the IoT fields, presenting a novel sensing paradigm for MEMS devices^31^. Traditional data collection processes, such as close-loop measurement and control for resonant accelerometers, can be replaced or act as a monitor reference. The RC system can directly handle target tasks, providing final outputs at the edge. The advancement presented in this paper sets a new benchmark for IoT devices, particularly in the area of edge computing, where direct processing of sensor data is crucial. Future work related to the topology and wearability of MEMS RC will be conducted to further prove its applicability in practical scenarios.

## Materials and methods

### Device fabrication

**Fig. 8:**
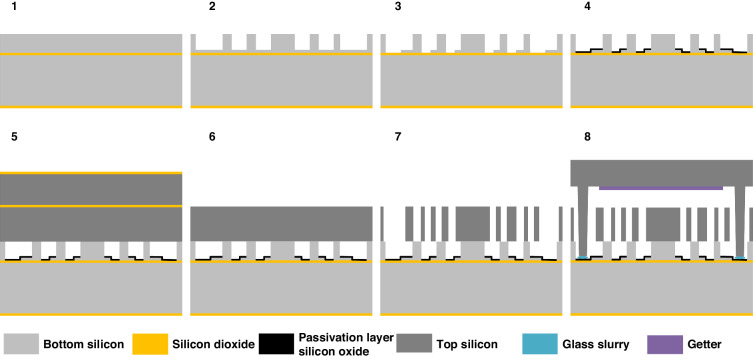
Fabrication processing flowchart. The process involves SOI wafer preparation, shallow trench patterning, patterning of bottom silicon and passivation layer silicon oxide, silicon-to-silicon bonding, BOX layer, and substrate silicon removal, top silicon electrode patterning, followed by glass frit wafer bonding and hermetic packaging

As shown in Fig. 8, the prototype device is constructed using silicon micro-manufacturing technology based on the standard Silicon on Insulator (SOI) micromachining process and a multilayer silicon wafer bonding procedure. The fabrication process initiates with a 6-inch SOI wafer (with a device thickness of 50 μm, an oxide thickness of 1 μm, and a substrate thickness of 380 μm) featuring a pre-etched shallow trench. The shallow cavity on the bottom SOI is patterned by photolithography and DRIE (deep reactive ion etching) techniques. The lower electrodes are delineated by patterned silicon, isolated from one another through etching to reveal the buried oxide (BOX) layer. Then deposit the oxidation layer to protect the bottom electrodes. Subsequently, a silicon-to-silicon bonding process is employed to affix the second SOI wafer, inverted, onto the pre-defined silicon electrodes following the deposition of silicon dioxide on the bonding plane. The BOX layer and the substrate silicon of the second SOI are then removed, and the top silicon electrodes are defined by photolithography and DRIE techniques. To achieve wafer-level hermetic packaging, a cap silicon wafer with etched cavities is bonded by glass frit, and getter material is deposited onto the aforementioned silicon dioxide bond plane, utilizing glass frit wafer bonding.

### Operation points tuning principles

Since the nonlinear region of the device in use is pivotal to RC performance, we offer principles to find the optimum operation point for our stiffness-modulated MEMS RC:

1. Position the accelerometer at zero-bias; set an initial bias V_dc_ and drive V_ac_; sweep the amplitude-frequency curve bidirectionally, as depicted in Fig. 5g, and find the smaller bifurcation point M (or the bigger one in the case of a softening spring).
2. Set the driving frequency f_d_ around point M; Sweep the amplitude-stiffness curve (via an acceleration series) bidirectionally as shown in Fig. 2b; ascertain whether the bifurcation point A is located in close proximity to the zero-bias (initial stiffness around 590 N/m in Fig. 2b).
3. Fine tune the f_d_ till point A approaches zero-bias, which is the standard of stiffness-modulated MEMS RC; if unsuccessful, revert to step (1) and fine tune the V_dc_ and V_ac_.
4. Repeat the preceding three steps until the standard is met.

### RC algorithm equations

We exclusively show equations of our new architecture. In the “input&reservoir” layer, the mapping of a nonlinear resonator is expressed as:

3 $$h(t)={{\mathrm{DF}}}\left(u(t)\right)$$

where DF represents the Duffing function from Eq. 1, u(t) is the input and h(t) is the resonator response. Following the readout, we obtain the sampled response h_i_ at the current time point i. In the output layer, the mask operator first introduces nonlinearities, that the original feature vector **x**_**i**_ is given by:

4 $${{\boldsymbol{x}}}_{{\boldsymbol{i}}}{\boldsymbol{=}}\left[{h}_{i}\,{{\cdot }}\,{\boldsymbol{m}},\,{{h}_{i}}^{2}\,{{\cdot }}\,{\boldsymbol{m}},\,{h}_{i}{h}_{i-j}\,{{\cdot }}\,{\boldsymbol{m}}\right]$$

where j is the recurrent time point and **m** is the mask with a length of N. Then, a delay brings feedback to **x**_**i**_ and a sigmoid function is applied, resulting in:

5 $${{\boldsymbol{r}}}_{{\boldsymbol{i}}}={{\mathrm{sig}}}{{\mathrm{moid}}}({{\boldsymbol{x}}}_{{\boldsymbol{i}}}+\alpha {{\boldsymbol{r}}}_{{\boldsymbol{i}}-{\boldsymbol{\tau }}})$$

where **r**_**i**_ is the final feature vector, τ is the delay length, and α is the feedback gain. Finally, **r**_**i**_ is concatenated with the previous vector and a bias term, getting the output vector **o**_**i**_, defined as:

6 $${\boldsymbol{o}}_{\boldsymbol{i}}=\left[[{\boldsymbol{r}}_{\boldsymbol{i}},\,{\boldsymbol{r}}_{{\boldsymbol{i}}-{\boldsymbol{k}}},\,{\boldsymbol{r}}_{{\boldsymbol{i}}-{\bf{2}}{\boldsymbol{k}}},\ldots ,\,{\boldsymbol{r}}_{{\boldsymbol{i}}-{\boldsymbol{sk}}}]//\gamma ,\,1\right]$$

where “//γ” denotes an even retention of elements with parameters γ, s, and k are positive integers. So, the length of **o**_**i**_ is 3N(s + 1)γ + 1. We employed ridge regression to train the output weight **w**, which is given by:

7 $${\boldsymbol{w}}={\bf{y}}{{\boldsymbol{O}}}^{{\boldsymbol{T}}}{\left({\boldsymbol{O}}{{\boldsymbol{O}}}^{{\boldsymbol{T}}}+\lambda {\boldsymbol{I}}\right)}^{-1}$$

where **y** is the ground truth, **O** is the feature matrix obtained by stacking up **o**_**i**_, λ is the regularization parameter and **I** is the identity matrix, and the current predicted output $\widehat{y}$ of the RC is given by:

8 $$\widehat{y}={\boldsymbol{w}}{{\boldsymbol{o}}}_{{\boldsymbol{i}}}$$

Equation 7 and Eq. 8 are presented for forecasting tasks. For classification tasks, the output weight is a matrix **W** consisting of several classifiers corresponding to each column, and the predicted output is a vector $\bf \widehat{y}$.

### TI-46 task

For this classification task, we utilized HNL, so the digital delay was unused. Here, we use N = 100, α = 0.4, j = 1, s = 1, k = 3, and γ = 50%. So, **r**_**i**_ contains 3N = 300 feature points and was then concatenated with the previous **r**_**i-3**_. Therefore, the length of **o**_**i**_ is 300 × 2 × 50% + 1 = 301, and the weight **W** has dimension (10 × 301). Regarding the winner-takes-all strategy, when the word “seven” is trained, the target is [0, 0, 0, 0, 0, 0, 0, 1, 0, 0]^T^. During testing, if the (m + 1)th element of the output is the largest and corresponds to the sought digit m, the correct number Y_m_ plus 1. The accuracy formula is expressed as:

9 $${\mathrm{ACC}}=\frac{\mathop{\sum }\nolimits_{m=0}^{9}{Y}_{m}}{C}$$

where ACC is the accuracy and C is the total test number.

### NARMA-10 task

The system behavior is governed by the following equation:

10 $$y\left(i\right)=0.3y\left(i-1\right)+0.05y\left(i-1\right)\mathop{\sum }\limits_{m=1}^{10}y\left(i-m\right)+1.5u\left(i-10\right)u\left(i-1\right)+0.1$$

The input u(i) is generated by randomly selecting values within the range of (0, 0.5). We took 1000 points for training and 500 points for testing. As ten adjacent data points are correlated in this forecasting task, we chose N = 50, α = 1.2, j = 10, s = 10, k = 1, and γ = 20%. So, each column in Fig. 7c is 3N = 150, and **o**_**i**_ is 150 × 11 × 20% + 1 = 331 in length, so as the weight **w**. We used NMSE for error evaluation, which is defined as:

11 $${{\mathrm{NMSE}}}=\frac{\mathop{\sum }\nolimits_{n=1}^{L}{\left({{y}}_{{n}}-\hat{{{y}}_{{n}}}\right)}^{2}}{L\,\cdot\, \mathrm{var}\left({\boldsymbol{y}}\right)}$$

where L is the total points, y is the ground truth, and $\widehat{y}$ is the prediction.
